# Antioxidant, Antibacterial and Dyeing Potential of Crude Pigment Extract of *Gonatophragmium triuniae* and Its Chemical Characterization

**DOI:** 10.3390/molecules27020393

**Published:** 2022-01-08

**Authors:** Ajay C. Lagashetti, Sanjay K. Singh, Laurent Dufossé, Pratibha Srivastava, Paras N. Singh

**Affiliations:** 1 National Fungal Culture Collection of India (NFCCI), Biodiversity and Palaeobiology Group, MACS’ Agharkar Research Institute, G.G. Agarkar Road, Pune 411004, India; lagashettiajay@gmail.com (A.C.L.); pnsingh@aripune.org (P.N.S.); 2 Faculty of Science, Savitribai Phule Pune University, Pune 411007, India; 3 CHEMBIOPRO Chimie et Biotechnologie des Produits Naturels, ESIROI Département Agroalimentaire, Université de la Réunion, F-97490 Sainte-Clotilde, Ile de La Réunion, France; 4 Bioprospecting Group, MACS’ Agharkar Research Institute, G.G. Agarkar Road, Pune 411004, India; psrivastava@aripune.org

**Keywords:** *Gonatophragmium triuniae*, pigments, bioactivity, dyeing, chemical characterization

## Abstract

Filamentous fungi synthesize natural products as an ecological function. In this study, an interesting indigenous fungus producing orange pigment exogenously was investigated in detail as it possesses additional attributes along with colouring properties. An interesting fungus was isolated from a dicot plant, *Maytenus rothiana*. After a detailed study, the fungal isolate turned out to be a species of *Gonatophragmium* belonging to the family *Acrospermaceae*. Based on the morphological, cultural, and sequence-based phylogenetic analysis, the identity of this fungus was confirmed as *Gonatophragmium triuniae*. Although this fungus grows moderately, it produces good amounts of pigment on an agar medium. The fermented crude extract isolated from *G. triuniae* has shown antioxidant activity with an IC_50_ value of 0.99 mg/mL and antibacterial activity against Gram-positive bacteria (with MIC of 3.91 μg/mL against *Bacillus subtilis*, and 15.6 μg/mL and 31.25 μg/mL for *Staphylococcus aureus* and *Micrococcus luteus*, respectively). Dyeing of cotton fabric mordanted with FeSO_4_ using crude pigment was found to be satisfactory based on visual observation, suggesting its possible use in the textile industry. The orange pigment was purified from the crude extract by preparative HP-TLC. In addition, UV-Vis, FTIR, HRMS and NMR (^1^H NMR, ^13^C NMR), COSY, and DEPT analyses revealed the orange pigment to be “1,2-dimethoxy-3*H*-phenoxazin-3-one” (C_14_H_11_NO_4_, *m/z* 257). To our understanding, the present study is the first comprehensive report on *Gonatophragmium triuniae* as a potential pigment producer, reporting “1,2-dimethoxy-3*H*-phenoxazin-3-one” as the main pigment from the crude hexane extract. Moreover, this is the first study reporting antioxidant, antibacterial, and dyeing potential of crude extract of *G. triuniae*, suggesting possible potential applications of pigments and other bioactive secondary metabolites of the *G. triuniae* in textile and pharmaceutical industry.

## 1. Introduction

Ascomycetous filamentous fungi are known to produce bio-pigments extensively used as colourants, additives, colour intensifiers, antimicrobial, antioxidants, etc., in different industries such as food, beverages, cosmetics, textiles, and pharmaceuticals [1,2,3]. Natural pigments/colours are gaining increased attention currently because of the adverse effects of synthetic colours on humans and the environment. Most of the synthetic colours have been found to be toxic, allergic, and carcinogenic to human beings, and hazardous to the environment [4,5,6]. This has increased the need for safe, natural, eco-friendly pigments as an alternative to synthetic pigments. Increasing demand for natural pigments necessitates a greater need to explore colours or pigments from safe, natural sources, especially from microbes (bacteria, fungi, algae, lichens, and actinomycetes). 

Fungi are currently emerging as a better and excellent source of natural pigments. Many researchers have reported fungi of different taxonomic groups exhibiting the production of pigments of diverse chemicals classes such as carotenoids, flavins, ubiquinones, anthraquinones, quinines, phenazines, etc. [1,7,8,9,10]. Due to the additional attributes of these “mycopigments” such as antimicrobial, anticancer, antioxidant, cytotoxicity, activity, etc. in addition to colouring property, they are being extensively used for a wide range of applications in food, textiles, medicines, paints, cosmetics, and electronics [8,9]. Some fungal pigments such as azaphilones, astaxanthin, Arpink Red, riboflavin, *β*-carotene isolated from *Monascus*, *Xanthophyllomyces dendrorhous*, *Penicillium oxalicum*, *Ashbya gossypii*, and *Blakeslea trispora*, respectively, are already in the market for their commercial and industrial applications [11]. Numerous studies have reported the dyeing potential of fungal pigments and suggested their possible use in the textile industry for dyeing different types of textile fabrics like cotton, silk, wool, etc. [7,8,12,13]. 

Literature indicates that extensive studies have been done worldwide on production, optimization, and applications of pigments from common ascomycetous fungi like *Monascus*, *Talaromyces*, *Aspergillus*, *Penicillium*, *Fusarium*, etc. [8,9]. Besides these conventional ones, several other genera, such as *Epicoccum*, *Trichoderma*, *Alternaria*, *Chaetomium*, etc., are reported to have good pigment production potential [7,14,15,16,17,18]; however, several genera of filamentous ascomycetes are still unknown and unexplored for their pigment production potential and exploitations. These unexplored fungi might prove to be a hidden treasure of novel bio-active pigments having a variety of applications.

Taking this view into account, we have planned the present research in which we have isolated an uncommon fungus, *Gonatophragmium triuniae,* from infected leaves of the *Maytenus rothiana*, a plant endemic to central Maharashtra (Western Ghats). Interestingly, it was found that this rare fungus produces a very good extracellular orange pigment on solid media [potato dextrose agar medium (PDA)] as well as in liquid media [potato dextrose (PD) broth]. For the characterization of pigment, pure culture of *G. triuniae* was subjected to flask level fermentation in PD broth, and pigments were extracted from the culture filtrate using Hexane and dried. The dried Hexane extract was then examined for its antimicrobial and antioxidant properties and also assessed for its dyeing potential on cotton fabric using two mordants (Alum & FeSO_4_). Finally, by preparative thin-layer chromatography (TLC), we purified an orange pigment from the crude pigment extract and identified it as “1,2-dimethoxy-3*H*-phenoxazin-3-one” based on ultra-violet (UV); Fourier transform infrared (FTIR); high-resolution mass (HRMS) spectroscopy; and ^1^H & ^13^C NMR, COSY, and DEPT analysis. 

Several natural and synthetic phenoxazines are well known for their bioactivity and dyeing properties. These phenoxazines were found to exhibit antioxidant, antibacterial, anti-proliferative, and anti-tumoral activities [19]. 3*H*-phenoxazin-3-one and its derivatives exhibiting numerous biological activities (antimicrobial, anticancer, antitumor, antiviral, antitubercular, anticoccidial, antineoplastic, phytotoxic, and cell growth-stimulating) have been reported from different microorganisms such as actinomycetes, lichens, and fungi [20]. Phenoxazine class of pigments such as Phenoxazone, Pycnosanguin, Cinnabarine, *O*-acetyl cinnabarine, 2-Amino-9-formylphenoxazone-1-carbonic acid, and 9-Hydroxymethyl-2-methylaminophenoxazone-1-carbonic acid methyl ester have been described from the fungus *Pycnoporus sanguineus* [21]. Similarly, Chandrananimycins A-C belonging to class 3*H*-phenoxazin-3-one, exhibiting antitumor activity against colon cancer (CCL HT29); breast cancer (LCL H460, CNCL SF268, MACL MCF-7); lung cancer (LXFA 526L, LXFL 529L); melanoma (MEXF 514L); and kidney tumor cells (PRCL PC3M, RXF 631L) has also been reported from *Actinomadura* sp. [22,23]. One of the recent studies evaluated the antibacterial activity of the synthesized derivatives of 3*H*-phenoxazin-3-one [20]. Based on the history of Phenoxazin class of pigments, their promising bioactivity, and their dyeing property, we may consider present compound 1,2-dimethoxy-3*H*-phenoxazin-3-one isolated from *G. triuniae* NFCCI 4873 as a good colourant as well as a potential candidate for application in the textile and pharmaceutical industry.

## 2. Results and Discussion

### 2.1. Morphological Identification

**Figure 1:**
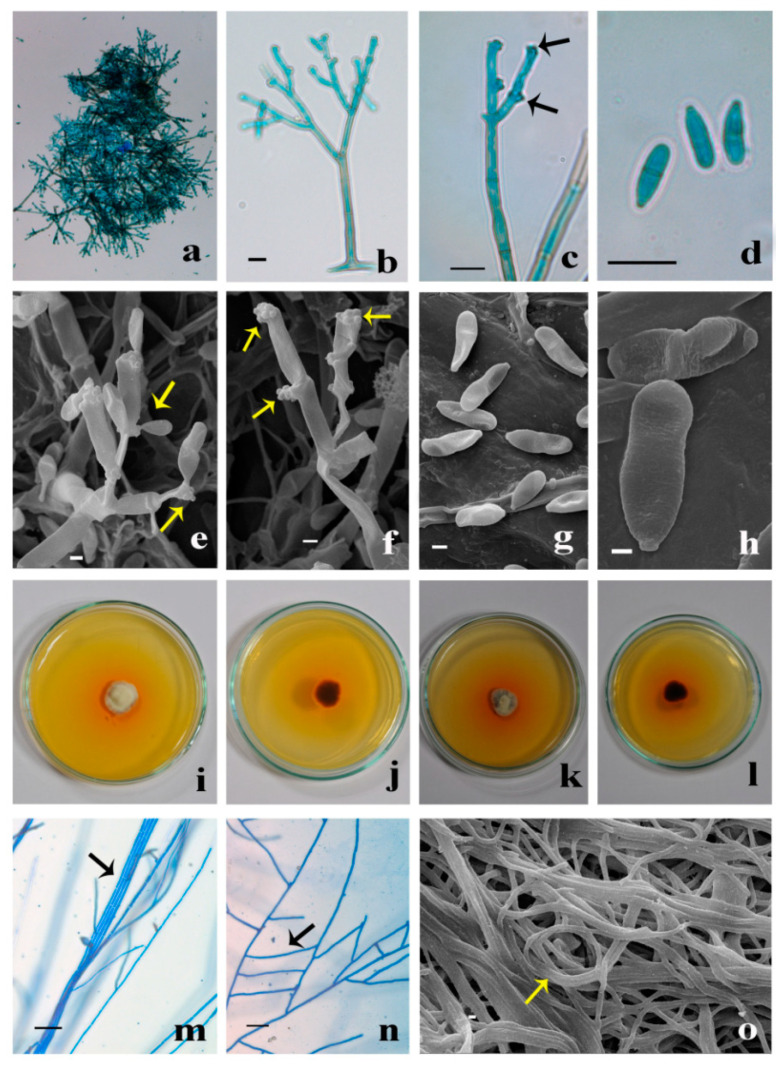
*Gonatophragmium triuniae* (NFCCI 4873): (**a**) Numerous conidiophores in low magnification; (**b**) Single dichotomously branched conidiophore; (**c**) Conidiophore and conidiogenous cells bearing dark scars (showing arrows); (**d**) Numerous conidia in high magnification; (**e**) SEM of conidiophores with attached conidia; (**f**) SEM of conidiophore bearing intercalary and terminal conidiogenous cells with dark scars (showing arrows); (**g**,**h**) SEM image of conidia; (**i**–**l**) Colonies of *G. triuniae* NFCCI 4873 on PDA and PCA (front and reverse view); (**m**,**n**) Hyaline sterile hyphal bundle and anastomosis in-vitro culture (showing arrows); (**o**) SEM of sterile hyphal bundles with coiling (showing arrow).

Leaf lesions, amphigenous, necrotic spots single or irregular in concentric rings, later spots unite to form large spots. Margin: greyish-white; center: white. Colonies hypophyllus, velvety, brown. Mycelium superficial. Hyphae branched, septate, pale olivaceous to subhyaline, smooth-walled, up to 6.5 µm wide. Stroma and hyphopodia are absent. Conidiophores arising from superficial hyphae, dichotomously branched, multi-septate (6–8), the width of conidiophore gradually decreasing towards the length; macronematous to mononematous, erect, smooth-walled, highly geniculate, nodose, basal half part of conidiophore olivaceous brown and subhyaline to light olivaceous towards the apex, 55–145 × 3.22–7 µm. Conidiogenous cells integrated, polyblastic, terminal to intercalary, swollen towards the apex, variable in size: 10–20 µm long, bearing 10–15 loci, scars thickened and darkened, dentate or plate-like about 1 µm diameter. Conidia solitary, dry, holoblastic, acropleurogenous, clavate, cylindrical, straight to slightly curved, 0–1 septate, smooth-walled, subhyaline to light olivaceous, base narrowly truncate, apex obtuse, hilum thickened and darkened, 6–15 × 2–3.6 µm (Figure 1).

Colonies on Potato Dextrose Agar (PDA), slow-growing, reach 26–29 mm diameter after 4 weeks of incubation at 25 °C; front view of colony light yellow (4A4), circular, raised, aerial mycelium slightly cottony, margin smooth. Reverse dark brown (7F8) with diffusible yellowish orange (5B8) pigment in entire media. Mycelium branched, septate, smooth-walled, with frequent anastomosis, hyaline, sterile. Colonies on Potato Carrot Agar (PCA), reach 27–28 mm diameter after 4 weeks at 25 °C; front view of colony grey (6C1), circular, raised, slightly cottony with margin smooth. Reverse dark brown (6F8) and with diffusible yellowish orange (5B8) pigment in entire media. Mycelium branched, septate, smooth-walled, anastomosis, hyaline, sterile (Figure 1).

### 2.2. Molecular Identification and Phylogeny

**Figure 2:**
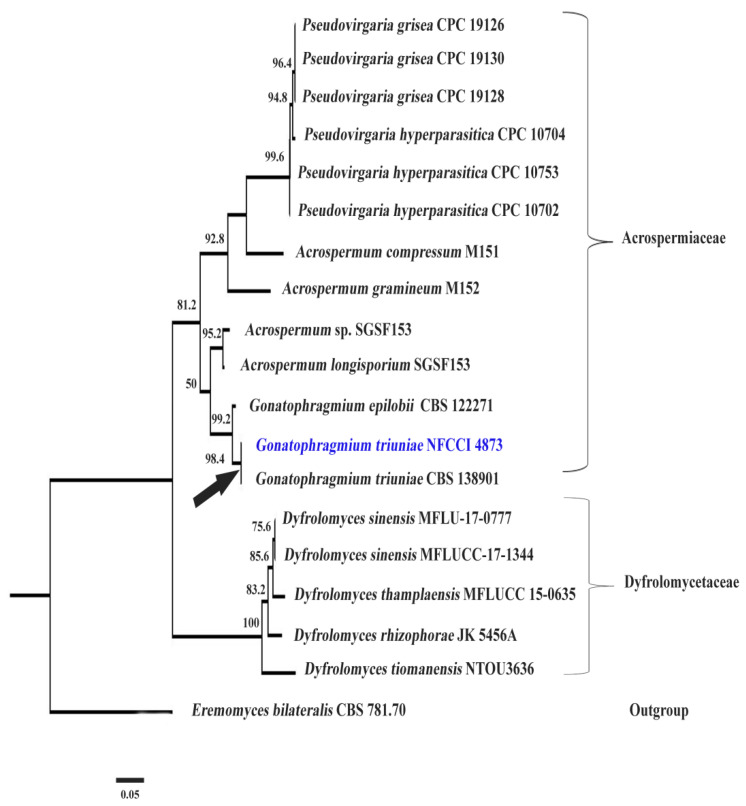
Phylogenetic tree of *G. triuniae* NFCCI 4873 based on the combined ITS & LSU rDNA sequence data. Digits on the nodes represent the likelihood bootstrap values.

Mega Blast analysis of ITS sequence of *G. triuniae* NFCCI 4873 showed 100% identity with type strain *G. triuniae* CBS138901; whereas LSU sequence showed 99.74% identity with *G. epiloblii* CPC 34889 and 99.61% similarity with *G. triuniae* CBS 138901. A phylogenetic tree was constructed based on combined ITS & LSU rDNA sequence data of a total of 19 genetically-related isolates, which shows that our isolate was clustered with *G. triuniae* CBS138901 with a very strong bootstrap value of 98.4 (Figure 2). Therefore, based on combined morphological and molecular phylogenetic analysis, the present isolate was identified as *G. triuniae*.

### 2.3. Analysis of Pigment Production on Different Media

**Figure 3:**
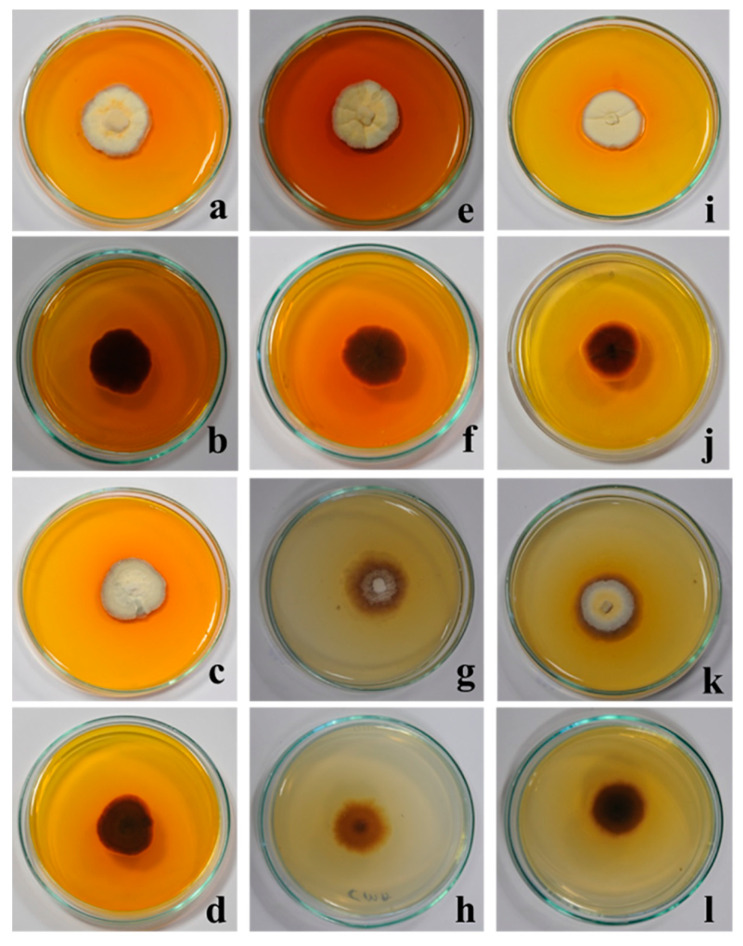
Studies on pigment production by *G. triuniae* (NFCCI 4873) on different media: (**a**,**b**) *G. triuniae* on PDA (front and reverse view); (**c**,**d**) *G. triuniae* on PCA (front and reverse view); (**e**,**f**) *G. triuniae* on SDA (front and reverse view); (**g**,**h**) *G. triuniae* on CMA (front and reverse view); (**I**,**j**) *G. triuniae* on CYA (front and reverse view; and (**k**,**l**) *G. triuniae* on CZA (front and reverse view).

After 4 weeks of incubation, *G. triuniae* NFCCI 4873 produced yellowish orange (5B8) pigment on potato dextrose agar (PDA), potato carrot agar (PCA), Sabouraud dextrose agar (SDA), and Czapek Yeast Extract Agar (CYA), which was completely diffused in media. In contrast, no pigment production was observed on cornmeal agar (CMA) and Czapek Dox agar (CZA) (Figure 3).

### 2.4. Pigment Production in Liquid Media

**Figure 4:**
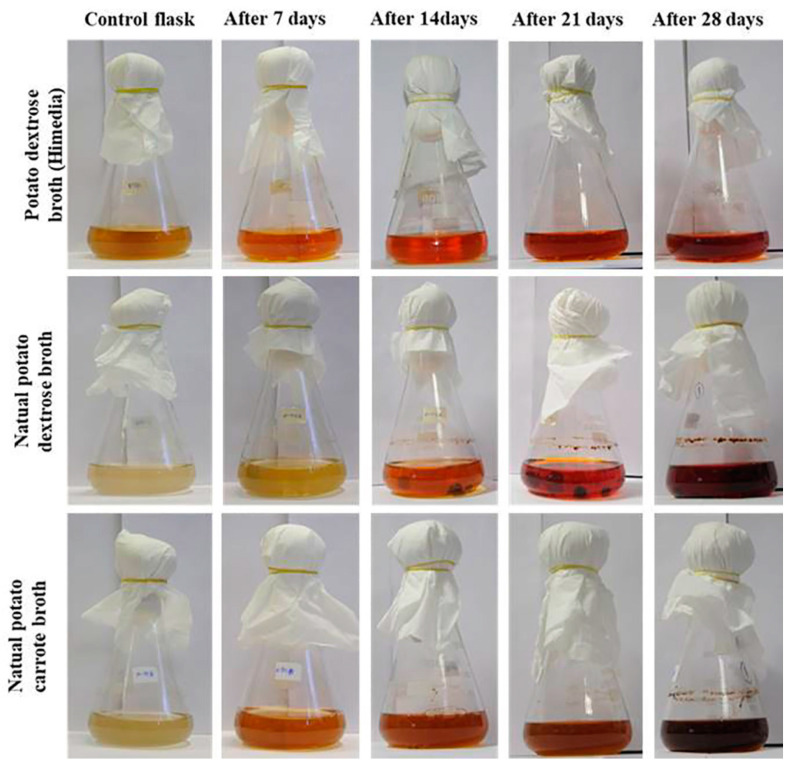
Analysis of pigment production by *G. triuniae* NFCCI 4873 in different liquid media.

**Figure 5:**
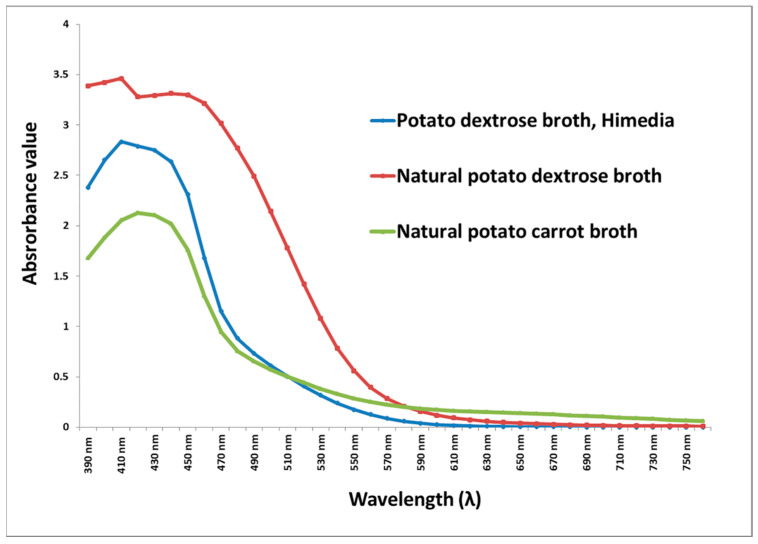
UV–Vis spectroscopy scanning (390–760 nm) of culture filtrates of different media.

*G. triuniae* NFCCI 4873 started pigment production earlier in PD broth of Hi-media compared to natural PD broth and natural PC broth. However, pigment production increased in natural PD broth and PC broth after 28 days of incubation compared to the PD broth of Hi-media (Figure 4). Scanning of coloured culture filtrate of *G. triuniae* NFCCI 4873 from three different media at a wavelength ranging from 390–760 nm shows that pigment production was higher in natural PD broth than PD broth (Hi-media) and natural PC broth (Figure 5). This clearly shows that natural potato dextrose broth supports the pigment production by *G. triuniae* NFCCI 4873 and suggests it as a good media for optimum pigment production.

### 2.5. Fermentation and Extraction of Pigments

**Figure 6:**
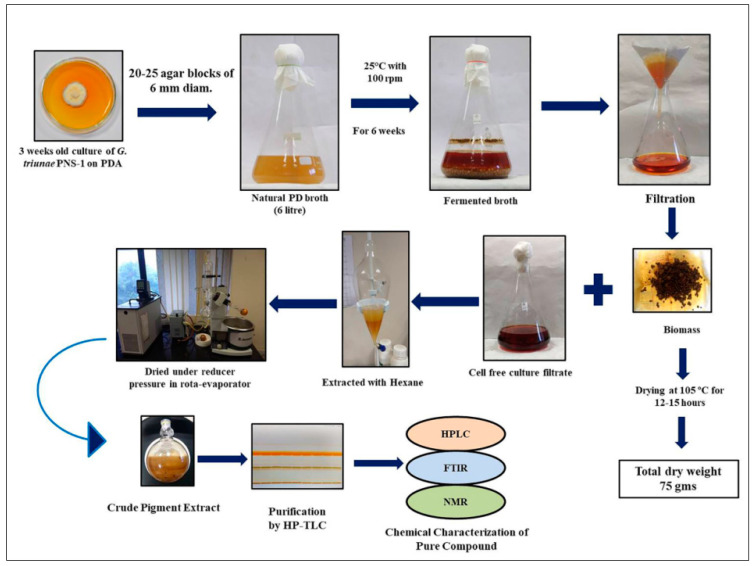
Schematic representation of fermentation, extraction, and purification of the compound from a pure culture of *G. triuniae* NFCCI 4873.

Upon filtration of 6-L fermentation broth of *G. triuniae* NFCCI 4873, approximately 4 L of coloured culture filtrate and 75 g of dry fungal biomass were obtained. Pigments from the coloured culture filtrate were extracted with Hexane, and the concentration of hexane extract in a rota evaporator under reduced pressure yielded 526.26 mg of crude hexane extract. This dried crude hexane extract was then used for subsequent analysis, testing, and purification (Figure 6).

### 2.6. UV–Vis Spectroscopy Analysis of Hexane Extract

**Figure 7:**
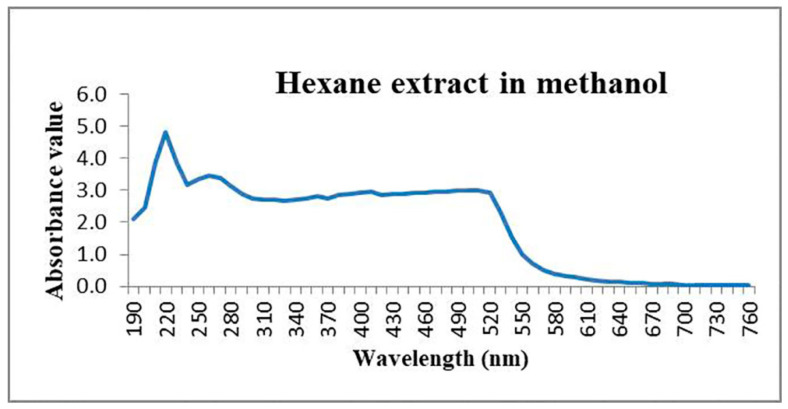
UV–Vis spectrum of hexane extract dissolved in methanol of *G. triuniae* NFCCI 4873.

Dried hexane extract dissolved in methanol showed maximum absorption at 220 nm (λ_max_) upon scanning at a wavelength ranging from 190–760 nm (Figure 7).

### 2.7. Antagonistic Activity Testing

**Figure 8:**
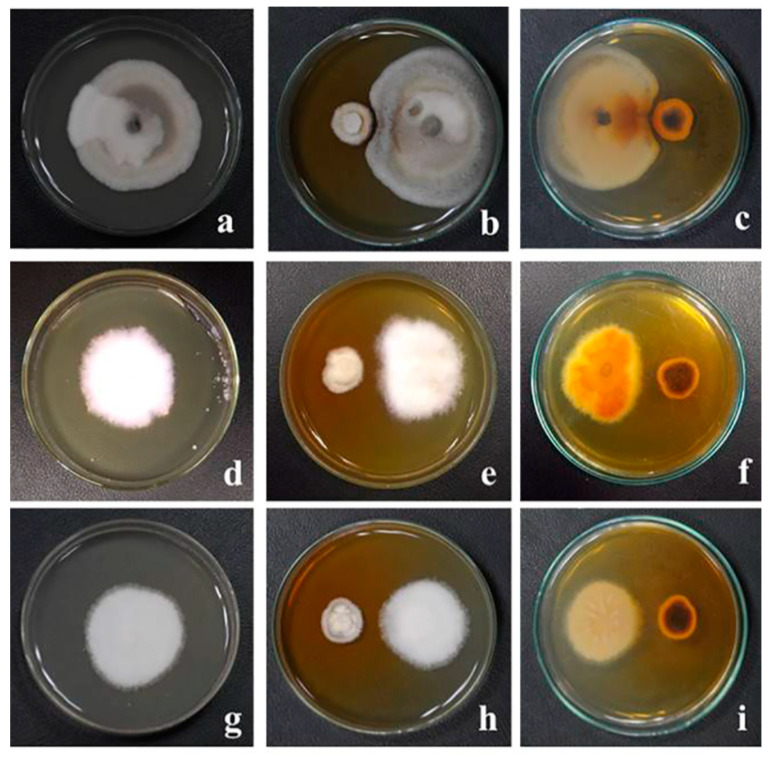
Antagonistic activity of *G. triuniae* NFCCI 4873: (**a**) *C. gloeosporioides* on PDA (control), (**b**,**c**) *G. triuniae* against *C. gloeosporioides* on PDA (front and reverse view), (**d**) *F. oxysporum* on PDA (control), (**e**,**f**) *G*. *triuniae* against *F. oxysporum* on PDA (front and reverse view), (**g**) *F. solani* on PDA (control), and (**h**,**i**) *G. triuniae* against *F. oxysporum* on PDA (front and reverse view).

The dual culture assay shows that *G. triuniae* NFCCI 4873 retarded the growth of fungal plant pathogens (*Colletotrichum gloeosporioides*, *Fusarium oxysporum*, and *Fusarium solani*). Among them, *G. triuniae* showed 30% inhibition of radial growth of *F. solani*, followed by 23% inhibition of radial growth of *C. gloeosporioides* and 16% inhibition of radial growth of *F. oxysporum*. This indicates that our isolate has the potential to inhibit the growth of other fungal plant pathogens (Figure 8).

### 2.8. Antioxidant Activity Testing of G. triuniae NFCCI 4873

**Figure 9:**
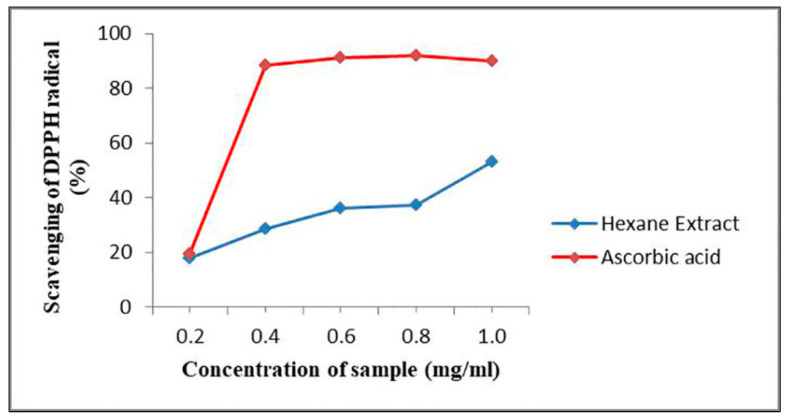
Scavenging effects of hexane extract of *G. triuniae* NFCCI 4873 on DPPH radical.

From the result of in-vitro antioxidant activity, it was observed that hexane extract of *G. triuniae* NFCCI 4873 showed satisfactory dose-dependent DPPH radical scavenging activity with an IC_50_ value of 0.99 mg/mL when tested with standard ascorbic acid (IC_50_ value of 0.24 mg/mL). This suggests the promising antioxidant potential of crude hexane extract of *G. triuniae* NFCCI 4873 (Figure 9).

### 2.9. Antimicrobial Activity Testing by Disc Diffusion

**Figure 10:**
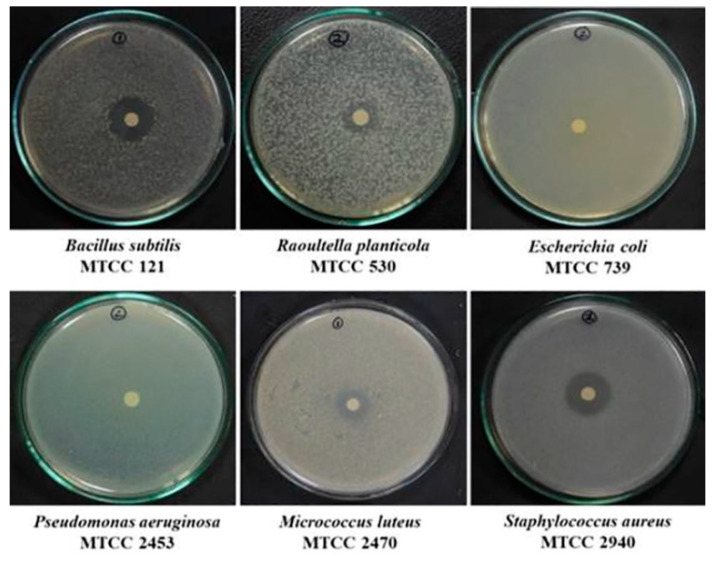
Antimicrobial activity of hexane extract of *G. triuniae* NFCCI 4873 against different test bacteria.

A crude hexane extract of *G. triuniae* showed promising antibacterial activity in the disc diffusion assay (Figure 10). The mean diameters of the inhibition zones of test strains for the crude hexane extract of *G. triuniae* NFCCI 4873 are shown in Table 1. From the results of disk diffusion assay, it was observed that crude hexane extract *G. triuniae* NFCCI 4873 showed promising antimicrobial activity mainly against Gram-positive bacteria (*B. subtilis*, *S. aureus*, and *M. luteus*), displaying an average zone diameter of 17.33 mm, 18.67 mm, and 17.33 mm respectively (Table 1). However, it showed very little activity against Gram-negative bacterium *R. planticola* with an average zone diameter of 12.33 mm; whereas no activity was observed against *E. coli* and *P. aeruginosa*. In general, *S. aureus* was more sensitive to the crude hexane extract, followed by *B. subtilis* and *M. luteus,* which show similar sensitivity to the crude hexane extract. The study revealed that crude hexane extract of *G. triuniae* NFCCI 4873 has potential antibacterial compounds that can be used in medicines for their possible applications as an antibiotic. 

### 2.10. MIC and MBC of Crude Pigment

**Figure 11:**
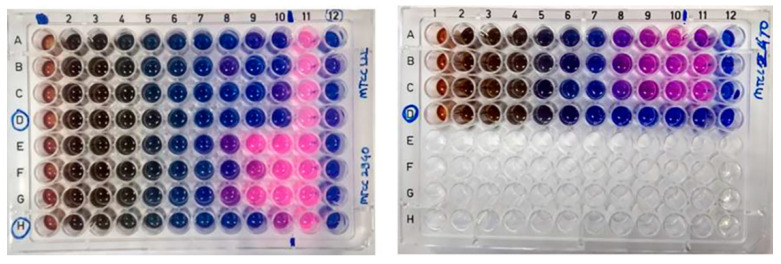
Microtitre plate showing MIC of crude pigment of *G. triuniae* NFCCI 4873.

The MIC of the crude hexane extract was found to be 3.91 μg/mL against *B. subtilis* and 15.6 μg/mL against *S. aureus*; whereas it was 31.25 μg/mL for *M. luteus*. The results of MIC show that *B. subtilis* had the lowest MIC, while the highest MIC obtained was for *M. luteus* (Figure 11).

The MBC of the crude hexane extract of *G. triuniae* NFCCI 4873 was found to be 1mg/mL against *B. subtilis* MTCC 121 and>1 mg/mL against *S. aureus* MLS 16 MTCC 2940; whereas it was 0.25 mg/mL against *M. luteus* MTCC 2470.

### 2.11. Dyeing of Cotton Fabric

**Figure 12:**
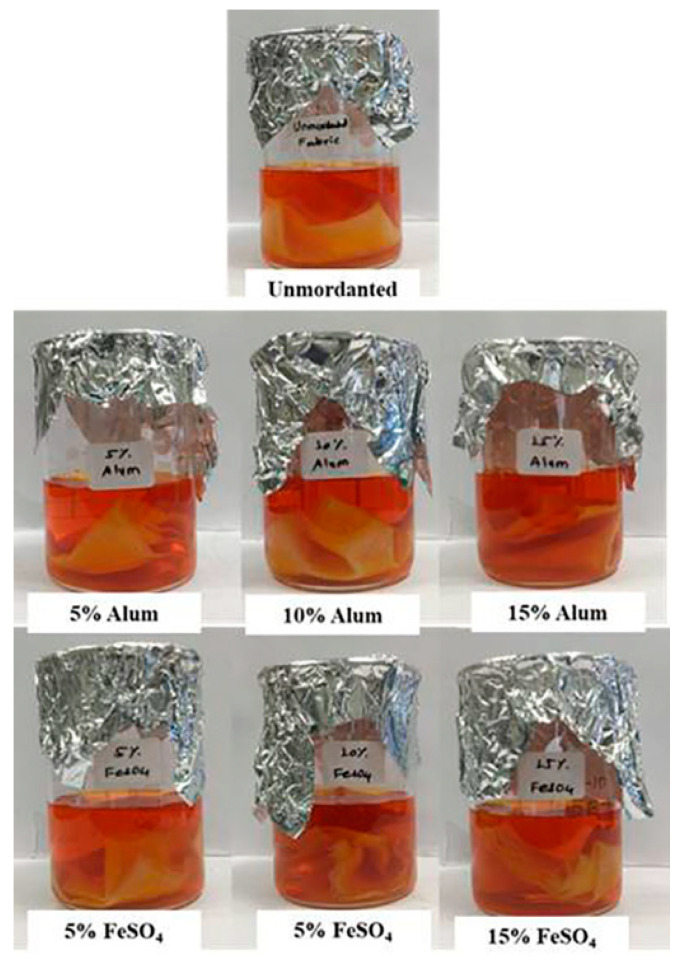
Dyeing of mordanted and un-mordanted cotton fabrics with crude pigment extract of *G. triuniae* NFCCI 4873.

**Figure 13:**
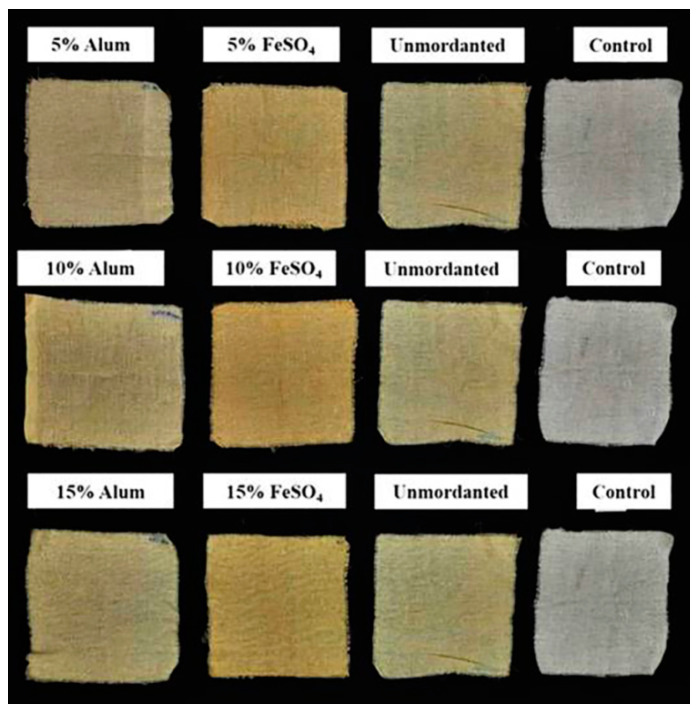
Cotton fabrics dyed with crude pigment extract of *G. triuniae* NFCCI 4873.

Results of the dyeing experiment showed that cotton fabrics mordanted with different mordants (FeSO_4_ and Alum) show more pigment uptake than un-mordanted fabric. Among the two mordants used, cotton fabrics mordanted with FeSO_4_ have shown more pigment uptake than cotton fabrics mordanted with different concentrations of Alum (Figure 12 and Figure 13). This clearly shows that pigments of *G. triuniae* NFCCI 4873 have potential applications in the textile industry for dyeing different textile fabrics. Moreover, previous studies have already reported phenoxazines and their derivatives as promising textile dyes. Their intense colours and chemical nature make them excellent vat dyes. Besides this, these dyes act as good colourants for paint, ink, papers, candles, soap, and plastic materials [24]. This confirms that the main pigment “1,2-dimethoxy-3*H*-phenoxazin-3-one” isolated and characterized from the present study may have promising dyeing potential in the textile industry.

### 2.12. GC-MS Analysis of Crude Hexane Extract

GC-MS analysis of crude hexane extract showed the peaks along with the mass of the organic molecules present in the extract. The chromatogram showed 25 peaks corresponding to the molecules (Figure S7). The detailed data of compounds present in the extract, their molecular weight, and respective retention time is presented in Table 2. Crude hexane extract majorly showed the presence of fatty acids [n-hexadecanoic acid and octadecanoic acid] and their derivatives, esters (glycidyl palmitate and dibutyl phthalate), and alkanes (hexatriacontane and undecane).

### 2.13. Purification of Pigment

**Figure 14:**
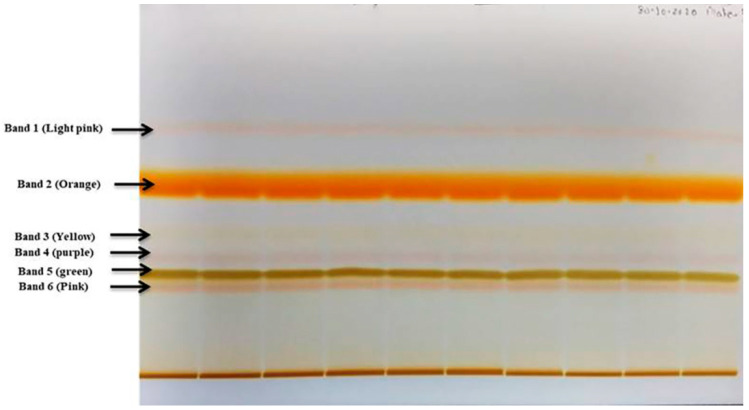
Separation of compounds in the crude pigment of *G. triuniae* NFCCI 4873 on TLC plate using HP-TLC.

Purification of crude pigment extract of *G. triuniae* NFCCI 4873 by preparative TLC yielded 40 mg of purified orange compound (band 2), which was named PNS-1-OR (Figure 14). This purified pigment was used for further chemical characterization for identification.

### 2.14. Chemical Characterization of Pure Compound (PNS-1-OR)

HRMS spectrum of the pigment gave a sodium adduct [M + Na]^+^ at *m/z* 280 (C_14_H_11_NO_4_Na) and molecular ion peak of C_14_H_11_NO_4_ at *m/z* 258 [M+1], suggesting C_14_H_11_NO_4_ as its molecular formula (Figure S2). The IR spectra showed a significant carbonyl (−C=O) peak at 1647 cm*^−^*^1^, (−C=N) 2331 cm*^−^*^1^, and aromatic C-H stretching at 2943 cm*^−^*^1^ (Figure S1). 

1,2-dimethoxy-3*H*-phenoxazin-3-one: This compound is orange solid. mp 145−150 °C; UV (MeOH) λ_max_ (log ε) 220 nm; IR (KBr) ν_max_ 2943, 2331, 1647 cm^−1^; ^1^H and ^13^C NMR data, see Table 3; HR-MS *m/z* 258 [M+1]^+^ (calcd. for C_14_H_11_NO_4_, *m/z* 257).

**Figure 15:**
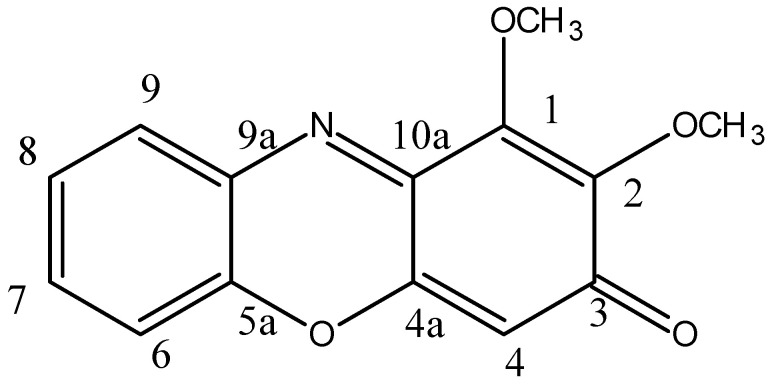
Natural product 1,2-dimethoxy-3*H*-phenoxazin-3-one.

The ^1^H NMR, ^13^C NMR details are shown in Table 3 along with the reported data and were found in agreement with the natural product, 1,2-dimethoxy-3*H*-phenoxazin-3-one (Figure 15) reported from *Acrospermum viticola*, a leaf spot fungus of Mulberry [25].

The pigment is isolated as an orange solid: yield (40 mg, 20%), mp 148–150 °C. The ^1^H NMR shows the distribution of protons signals between 1.0 and 8.0 ppm. Analysis of the ^1^H NMR (Figure S3) and 2D-COSY spectra (Figure S4) revealed a sequence of 11 total hydrogens at *δ* 4.12 (s, 3H, OCH_3_), 4.14 (s, 3H, OCH_3_), 6.23 (s, 1H, 4-H), 7.33 (dd, 1H, *J* = 8.1, 1.07 Hz, 6-H), 7.39 (td, 1H, *J* = 7.71, 1.37 Hz, 8-H), 7.54 (td, 1H, *J* = 7.7, 1.37 Hz, 7-H), and 7.92 (dd, 1H, *J* = 7.9, 1.53 Hz, 9-H). The ^1^H NMR of **1a** showed two singlets at *δ* 4.11 (s, 3H, OCH_3_) and *δ* 4.14 (s, 3H, OCH_3_), revealing two methoxy groups at positions one and two. A singlet was observed at *δ* 6.23 (s, 1H, C-**4**) next to the carbonyl group at position four. The characteristic signals for aromatic protons were observed at *δ* 7.33 as a doublet for (dd, *J* = 8.09 Hz, 1H, 6-H), triplet at *δ* 7.39 (td, *J* = 7.71Hz, 1H, 8-H), triplet at *δ* 7.54 (td, *J* = 7.70, 1H, 7-H), and doublet at *δ* 7.92 (dd, *J* = 7.93 Hz, 1H, 9-H). The ^1^H COSY spectrum showed the correlation of a 6-H proton at δ 7.33 with a 7-H proton at δ 7.54 only. The 8-H proton at *δ* 7.39 showed a correlation with 7-H and 9-H at *δ* 7.54 and 7.92, respectively. The 7-H proton at *δ* 7.54 displayed a correlation with 6-H and 8-H at *δ* 7.33 and 7.39, respectively. The 9-H proton at 7.92 displayed a correlation with 8-H at *δ* 7.39.

The ^13^C spectrum of PNS-1-OR was observed between 60 ppm and 200 ppm. The ^13^C NMR spectra (Figure S5) showed 14 carbons at *δ*_C_ 61.1 (OCH_3_), 62.2 (OCH_3_), 104.6 (C-**4**), 115.9 (C-**6**), 125.3 (C-**8**), 130.3 (C-**9**), 132.2 (C-**7**), 132.6 (C-**9a**), 143.4 (C-**5a**), 145.2 (C-**1**), 145.8 (C-**2**), 147.2 (C-4a, **10a**), and 181.8 (C-**3**). The DEPT 135 analysis (Figure S6) showed the carbons that are attached to hydrogens. Therefore, peaks observed at δ 61.22 and 62.26 belong to two methoxy carbon. The peak detected at δ 104.65 corresponds to C-**4**. The displayed peaks at *δ* 115.98, 125.375, 130.38, and 132.21 belong to C-**6**, C-**8,** C-**9**, and C-**7**, respectively.

The elemental analysis of CHN for the formula C_14_H_11_NO_4_ was obtained as C 64.97, H 4.29, and N 5.44%, which was found in agreement with the calculated values C 65.37, H 4.31, and N 5.44%. It was confirmed that the isolated pigment is 1,2-dimethoxy-3*H*-phenoxazine-3-one.

## 3. Materials and Methods

### 3.1. Collection and Isolation of Fungus

Infected leaves of *Maytenus rothiana* were collected in sterile paper bags from the Western Ghat region (Mahabaleshwar), Maharashtra, India. Collected samples were transported to the laboratory and stored in a refrigerator at 4 °C till their processing. Collected leaves (infected with fungus) were used to isolate fungus. The lower leaf surface was found to be colonized by fungus. For the in-vitro culture of fungus, spore mass was lifted with the help of a fine needle from the infected leaf surface and suspended in 1 mL sterile distilled water incorporated with Tween 20. Then, with a micropipette’s help, 200 μL of spore suspension was spread on a 2% Neutral agar plate using a spreader, and the plate was incubated at 25 °C overnight. On the next day, the plate was observed under the CX-21 compound microscope, and germinated single spores with agar block were picked up with the help of a sterile needle and transferred on sterile potato dextrose agar (PDA) plates. Plates were incubated at 25 °C for 7 days. The pure growing colonies were further sub-cultured on fresh PDA plates and slants. Slants were stored in a refrigerator at 4 °C till further use.

### 3.2. Morphological Identification and Deposition of Fungal Culture

Necrotic lesions on the infected leaves were marked. Scrape mount slides were prepared in lactophenol cotton blue and observed under a microscope; based on the literature, the fungus was identified as *Gonatophragmium* sp. Similarly, slides were prepared in lactophenol cotton blue mount from axenic culture. Microphotographs were taken using Carl Zeiss AXIO-10 microscope, and scanning electron microscopic (SEM) images were taken using images ZEISS EVO MA 15 Scanning electron microscope at 20 KV. 

A pure culture of *G. triuniae* was inoculated on potato dextrose agar (PDA) and potato carrot agar (PCA) to study cultural and microscopic characters. Plates were incubated at 25 °C for 14 days, and cultural and microscopic characters were noted upon completion of incubation. Slide culture [26,27] and grass leaf technique [28] were used to get the sporulation, but no sporulation was observed. 

Live and pure fungal culture of *G. triuniae* were deposited in the National Fungal Culture Collection of India (NFCCI), Agharkar Research Institute, Pune, under the accession number NFCCI 4873. Voucher culture *G. triuniae* NFCCI 4873 was deposited in Ajrekar Mycological Herbarium (AMH), Agharkar Research Institute, Pune, with accession number AMH 10289.

### 3.3. Molecular Identification and Phylogeny

For the identification and authentication of fungal culture up to species level, molecular characterization was done. The fungal genomic DNA was isolated following the standard protocol [29]. Then by polymerase chain reaction (PCR), the ITS (internal transcribed spacer) and LSU (large subunit) regions of rDNA were amplified from the extracted genomic DNA using the primers ITS-4 & ITS-5 [30] and LR-0R & LR-7 [31], respectively. FavorPrepTM PCR Purification Kit (Favorgen, Biotech Corporation, Taiwan) was used to purify PCR products. Purified PCR products were subjected for sequencing by the BigDye Terminator v3.1 Cycle Sequencing Kit (Applied Biosystems, Waltham, MA, USA) and ABI Avant 3100 automated DNA sequencer (Applied Biosystems, USA). The manually edited sequences of ITS and LSU regions of rDNA of our fungal isolate were deposited in the nucleotide sequence database of NCBI (Gene Bank Accession Numbers: ITS- MW193329 and LSU- MW144438). 

The ITS and LSU rDNA sequences of our fungal isolate were subjected to Mega BLASTn sequence homology searches. Based on the BLASTn search results, genetically related species, including genus *Gonatophragmium*, *Acrospermum*, *Pseudovirgaria*, and *Dyfrolomyces*, were chosen to construct the phylogenetic tree (Table 4). Phylogenetic analysis of *G. triuniae* NFCCI 4873 was performed based on a combined ITS and LSU rDNA sequence data of a total of 19 fungal cultures. The *Eremomyces bilateralis* CBS 781.70 was chosen as an out-group. With the help of the MUSCLE algorithm, multiple sequence alignment was performed in MEGA 7 [32]. Phylogenetic tree of *G. triuniae* NFCCI 4873 was constructed based on combined data of ITS & LSU rDNA sequences in IQ-TREE multicore version 1.6.11 [33] using the Maximum Likelihood method with best-fit model TN+F+G4. Selection of the best-fit model was done using the ModelFinder employed in IQ-TREE.

### 3.4. Analysis of Pigment Production on Different Media

A pure culture of *G. triuniae* NFCCI 4873 was inoculated on different media such as potato dextrose agar (PDA), potato carrot agar (PCA), Sabouraud dextrose agar (SDA), Czapek Dox agar (CZA), cornmeal agar (CMA), and Czapek Yeast Extract Agar (CYA) in duplicates and incubated at 25 °C for 28 days to assess the pigment production potential. After incubation, the colour of the pigment diffused in media was recorded using the Methuen handbook of colour [34].

### 3.5. Analysis of Pigment Production in Liquid Media

The culture of *G. triuniae* was also tested for its pigment production ability in different liquid media such as potato dextrose broth (PDB, Hi-media), natural potato dextrose broth (n-PDB), and natural potato carrot broth (n-PCB). Four agar blocks of a pure culture of *G. triuniae* (6 mm diameter) from 20-days-old PDA culture plate were inoculated in a 250 mL Erlenmeyer flask containing 100 mL media (each media in the separate flask), and flasks were incubated at 25 °C with 150 rpm. All tests were performed in duplicates. All flasks were observed intermittently after every week from the date of inoculation for pigment production, and observations were noted down. After 4 weeks of incubation, culture broths were filtered, and absorption spectrum analyses of the coloured filtrates were performed using a UV–VIS spectrophotometer (Shimadzu UV-2450). The absorbance of coloured culture filtrates was recorded in the visible light range from 390–760 nm with a 10 mm optical pathlength and 0.1 nm resolution.

### 3.6. Fermentation and Extraction of Pigments

*G. triuniae* culture was subjected to flask scale fermentation in a total of 6 L (four flasks containing 1.5 L of media) of natural potato dextrose broth (PD broth). Each flask was inoculated with 20–25 mycelial disks (6 mm diameter) of *G. triuniae* from 3-weeks-old PDA culture plate using a cork borer and incubated at 25 °C with 100 rpm for 4–6 weeks. After incubation, the coloured culture broth was filtered through pre-weighed blotting paper, and culture filtrate was collected in a separate flask. Later, the pigments from the culture filtrates were extracted thrice with an equal volume of Hexane. With the help of a separating funnel, the Hexane part was separated from the culture filtrate. The separated hexane part was evaporated to dryness under reduced pressure in a rota evaporator (Heidolph, Schwabach, Germany). The resulting concentrated hexane extract was used for further experiments. Finally, biomass collected in a pre-weighed blotting paper was dried at 105 °C for 12–15 h and weighed to measure the yield of biomass concentration [35].

### 3.7. UV–VIS Spectroscopy Analysis of Hexane Extract

UV-Visible spectroscopic analyses of the crude hexane extracts were performed using a UV-VIS spectrophotometer (Shimadzu UV-2450). The crude hexane extract was evaporated to dryness and then dissolved in methanol solvent, and absorbance was recorded in the range of 190–760 nm with a 0.1 nm resolution and 10 mm optical path length. 

### 3.8. Antagonistic Activity of G. triuniae NFCCI 4873

Antagonistic activity of *G. triuniae* against three fungal pathogens (*Colletotrichum gloeosporioides*, *Fusarium oxysporum* & *Fusarium solani*) was evaluated by dual culture technique [36]. Mycelial disks of 6 mm diameter were excised from the edge of actively growing culture of *G. triuniae* and fungal pathogens and inoculated on opposite ends of PDA plates equidistant from the periphery (each pathogen separately with test culture). For control, PDA plates were inoculated with a pathogen without test culture. Plates were then incubated at 25 °C for 14 days. Experiments were performed in duplicates. After completion of incubation, the radial growth of each fungal pathogen on PDA plates was measured, and percentage inhibition of radial growth (PIRG) of fungal pathogens was calculated relative to the control plate using the following formula:

PIRG = [(R_1_ − R_2_)/R_1_] × 100

where R_1_ is the radial growth of the fungal pathogen in the control plate, and R_2_ is the radial growth of the pathogen in the presence of test culture (*G. triuniae*) [37].

### 3.9. In-vitro Antioxidant Activity of Crude Pigment

In-vitro antioxidant activity of hexane extracts was tested using DPPH radical scavenging method [38]. Different concentrations (0.2, 0.4, 0.6, 0.8, and 1.0 mg/mL) of the extract and standard solution (Ascorbic acid, Sigma, USA) were used, and for that, dilutions were prepared in methanol. For the assay, 10 μL of extract or standard solution was added to 200 μL of 0.1 mM DPPH in methanol solution in a 96-well microtitre plate (Thermofisher, Waltham, MA, USA). All reactions were performed in triplicates. The plate was then incubated at 37 °C for 30 min in the dark. After incubation, the absorbance of the solution in each well was measured at 490 nm using a Synergy HT Multi-detection microplate reader [BioTek, Winooski, VT, USA]. The percentage of radical scavenging activity of the hexane extract was calculated by the following formula:

DPPH radical scavenging activity (%) = [(OD control − OD sample)/OD control] × 100

where OD means optical density or absorbance value. The IC_50_ value (concentration of sample required to scavenge 50% of free radicals) of hexane extract was determined.

### 3.10. Antimicrobial Activity of Crude Hexane Extract

Crude pigment sample was screened against a panel of test organisms, including *Escherichia coli* (MTCC 739), *Bacillus subtilis* (MTCC 121), *Staphylococcus aureus* MLS 16 (MTCC 2940), *Pseudomonas aeruginosa* (MTCC 2453), *Raoultella planticola* (MTCC 530), and *Micrococcus luteus* (MTCC 2470). The test strains were procured from Microbial Type Culture Collection (MTCC), CSIR-Institute of Microbial Technology (IMTECH), Chandigarh, India. Ciprofloxacin hydrochloride (1 mg/mL), vancomycin hydrochloride (1 mg/mL), chloramphenicol (1 mg/mL), streptomycin (1 mg/mL), and ampicillin (1 mg/mL) against bacteria, were used as positive controls.

### 3.11. Antimicrobial Activity by Disc Diffusion Method

The antimicrobial activity of the hexane extract of *G. triuniae* was tested using the disk diffusion method. The standardized microbial inoculum of the test strain was prepared in a saline solution (~10^6^–10^8^ CFU/mL) from the 24 h old culture plates. The culture media used was Muller-Hinton agar (MHA). A direct colony suspension method was used for the inoculum preparation in which well-isolated colonies from 24 h old culture plates were selected and suspended in sterile saline. Then saline suspensions of the test cultures were adjusted to achieve turbidity equivalent to a 0.5 McFarland standard. This was done by adjusting the turbidity of the cell suspensions between 0.08 and 0.12 AU in a UV-Vis Spectrophotometer (Shimadzu UV-2450) at 625 nm as recommended by CLSI guidelines. This results in a suspension with approximately 1–2 × 10^8^ colony-forming units (CFU)/mL for *Escherichia coli* ATCC^®^a 25922. The resulted suspension was added in sterile molten Muller-Hinton agar (0.5 mL/100 mL of media). The final concentration of the cells in the media was 0.5–1.0 × 10^8^ CFU/mL. Then media was poured in Petri plates and allowed to solidify [39]. 

Sterile Whatman filter paper discs impregnated with known amounts of the test sample (1mg/mL of dried hexane extract dissolved in methanol) were placed on the surface of an agar plate that was inoculated with a standardized suspension of microorganisms that were to be tested. Standard antibiotics like ampicillin (1 mg/mL), ciprofloxacin hydrochloride (1 mg/mL), streptomycin (1 mg/mL), vancomycin hydrochloride (1 mg/mL), and chloramphenicol (1 mg/mL) were used as positive controls. Paper discs impregnated with only methanol and sterile water were used as negative controls. Plates were left at room temperature for 1–2 h for the diffusion of samples and then incubated at 37 °C for 24 h. After completion of incubation, all plates were observed for the zone of inhibition. The diameters of the zone of inhibition were measured in millimeters, including the disk diameter (6 mm). All experiments were performed in triplicates.

### 3.12. Determination of Minimum Inhibitory Concentration (MIC) and Minimum Bactericidal Concentration (MBC)

#### 3.12.1. Preparation of Pigment Stock

Forty milligrams of crude pigment was dissolved in 1 mL of DMSO in a 2 mL Eppendorf tube, and a tube was mixed well. Then this 1 mL solution was then diluted 10 times in sterile Muller–Hinton broth (MHB), and the resultant solution was stored in the refrigerator at 4 °C until further use. 

#### 3.12.2. Preparation of Standardized Inoculum

The standard inoculum (0.5 McFarland) of Gram-positive bacteria (*Bacillus subtilis*, *Staphylococcus aureus* MLS 16, and *Micrococcus luteus*) was prepared by the direct colony suspension method as recommended by CLSI guidelines in which the OD_625_ value was adjusted to the equivalent of 10^8^ CFU/mL in a Shimadzu UV-2450 UV–VIS spectrophotometer [39]. 

#### 3.12.3. MIC and MBC Experiment 

The minimum inhibitory concentration (MIC) of the crude pigment extract was assessed using the standard method [39]. The MIC of the crude pigment extract was determined by the broth microdilution method in a 96-well plate as per the CLSI recommended protocol. All the wells of the microtitre plate from columns 2–11 were added with 50 μL of sterile Muller–Hinton broth (MHB). The last (12^th^) column was added with 100 μL of MHB as sterility control. One hundred microlitres of pigment solution (2 mg/mL) was added to the first column of the 96-well microtitre plate. Then, using a micropipette, a two-fold serial dilution was performed by transferring 50 μL of the pigment solution from the first well to the succeeding well and up to the 10^th^ well, and the final 50 μL of the solution was discarded. Standardized inoculums of bacteria were diluted 1:150 times in sterile MH broth to get 10^6^ CFU/mL concentrations of bacteria. Then, 50 μL of microbial suspension (10^6^ CFUs/mL) was added in each well from well 2–11; while in rows D and H, from well 1–10, there was no addition of bacterial suspension, which were treated as pigment control. Plates were incubated at 37 *°*C for 20 h. Each test was performed in triplicates.

After incubation at 37 °C for 20 h, 30 μL of 0.01% resazurin was added to each well, and then plates were further incubated for 2 h at 37 °C for the change of colour. The well-containing lowest concentration of pigment showing no colour change was considered as the MIC value.

After 20 h of incubation at 37 °C, 10 μL solution from each well (2–11) of the 96-well microtitre plate was plated on Muller–Hinton agar plate, and plates were incubated at 37 °C for 24 h. After completion of incubation, plates were observed for the growth of bacteria. The lowest concentrations that completely kill the bacteria and do not show growth on the MHA plate were considered minimum bactericidal concentration (MBC).

### 3.13. Dyeing of Cotton with a Crude Pigment of G. triuniae NFCCI 4873

#### 3.13.1. Textile Fabric

Raw cotton fabric was collected from the local market, Pune. Cotton fabric was then cut into 10 × 10 cm pieces (each weighing 1 gm) and used for the dyeing experiment.

#### 3.13.2. Scouring

Twelve cotton fabric pieces of 10 × 10 cm (12 g) were pre-soaked in milli-Q water and then cooked in 2.5 L of Milli-Q water containing 20 mL of non-ionic detergent (Triton-X-100) for 1 h at 70 °C in a water bath to remove oil and dirt. Scoured cotton fabrics were then rinsed thoroughly with running water and air-dried [40].

#### 3.13.3. Mordanting

Scoured cotton fabric pieces were mordanted by the pre-mordanting technique [40,41]. Cotton fabric pieces (10 × 10 cm) were mordanted with different concentrations (5%, 10%, and 15% *w/w* of fabric) of Alum and FeSO_4_ for 45 min at 70 °C with a 1:20 material to liquid ratio (MLR). After completing mordanting, fabric pieces were rinsed in running water and finally allowed to air dry.

#### 3.13.4. Preparation of Dye Bath

One hundred milligrams of dried crude pigment of *G. triuniae* NFCCI 4873 was re-dissolved in 500 mL of Milli-Q water using a magnetic stirrer. The resultant coloured solution was used as a dye bath for dyeing cotton fabric.

#### 3.13.5. Dyeing

Unmordanted and pre-mordanted cotton fabric pieces were dyed with a crude pigment solution of *G. triuniae* NFCCI 4873. Cotton fabric pieces (10 × 10 cm) were dyed at a material to liquor ratio (MLR) of 1:50 at 70 °C for 45 min in a water bath. The pH of the dye bath was not controlled. Dyed fabric pieces were then treated with 1% acetic acid and washed thoroughly in running water. The dyed fabric pieces were then rinsed with cold water and dried overnight in the shed [40].

### 3.14. GC–MS Analysis

The dried hexane extract was subjected to derivatization using *N*-Methyl-*N*-(trimethylsilyl)trifluoroacetamide [MSTFA] as a silylating agent. For derivatization, about 5 mg of sample was dissolved in 100 μL of pyridine in a glass vial followed by the addition of 100 μL of MSTFA. The solution was mixed well, heated at 60 °C for 20 min, and cooled to room temperature. After derivatization, the sample was subjected to gas chromatography-mass spectrometric analysis using GCMS-TQ8030 (Shimadzu, Nakagyo-ku, Kyoto, Japan). A fused silica column [RTX-5MS (30 m × 0.25 mm × 0.25 µm)] was used. The temperature of the column was programmed from 50 to 280 °C at a rate of 10 °C/min. The injection port temperature was set at 280 °C, and a split ratio of 1:40 was used for the analysis. Helium was used as the carrier gas at a flow rate of 1.0 mL/min. Electron ionization source of 70 eV and a mass range of *m/z* 35–800 U was used for MS detection. The resultant MS peaks in the GC-MS chromatogram were identified by comparing and matching the mass and mass fragmentation pattern with the reference mass and mass fragmentation pattern in the NIST05 MS library.

### 3.15. Purification of a Compound by HP-TLC

Two-hundred milligrams of crude pigment extract of *G. triuniae* NFCCI 4873 was dissolved in 4 mL of acetone, and compounds present in crude extract were separated on silica plates using HP-TLC (CAMAAG). Hexane:ethyl Acetate (40:60) was used as the mobile phase; thin aluminium silica plates (Merck) were used as the stationary phase. After separating compounds through HP-TLC, the orange band separated on plates was cut, and the compound attached to silica was extracted with acetone. Acetone extracts were collected in a round bottom flask and dried using a rota evaporator under reduced pressure using Heidolph rota evaporator. Finally, the weight of the dried, purified pigment was measured and recorded.

### 3.16. Chemical Characterization of Purified Orange Compound (PNS-1-OR)

The high-resolution mass spectrum (HRMS) of the pure orange pigment labeled as PNS-1-OR was recorded on a Bruker IMPACT HD. FTIR spectrum of pigment was recorded on a Shimadzu-IRAffinity-1 FTIR spectrophotometer in the frequency range 4000−400 cm^−1^. Pure compound was subjected to ^1^H (500 MHz) and ^13^C (125 MHz) NMR in Bruker 500 MHz NMR instrument using CDCl_3_ as a solvent for dissolving sample and TMS as internal standard. Chemical shifts (*δ*-values) are given in parts per million (ppm), and the coupling constants (*J*-values) are given in hertz (Hz).

## 4. Conclusions

Many fungi of different taxonomic groups producing a wide variety of pigments of different colours and chemical classes have been reported by researchers across the world. Among them, some fungal pigments find their application in different industries possessing promising colouring properties. The present study also reports one of the unconventional fungi, i.e., *G. triuniae,* showing very good pigment production potential. Moreover, this is the first experimental work reporting pigments and other secondary metabolites from the fungus *G. triuniae*. Based on the results of the antibacterial activity of the crude pigment extract, we conclude that the crude pigment extract shows the presence of antibiotic compounds, exhibiting antibacterial activity against Gram-positive bacteria. In addition to this, the DPPH radical scavenging activity of the crude pigment extract confirmed the presence of antioxidant compounds in the crude pigment extract. Such bioactivities (antibacterial and antioxidant) of the crude pigment extract have elevated the *G. triuniae* as a promising source of bioactive compounds for their possible use in the medicine and pharmaceutical industry. Besides this, the dyeing property of the crude pigment extract revealed the potential use of pigments of *G. triuniae* in the textile industry for dyeing different types of fabrics.

The purification of crude pigment extract of *G. triuniae* finally yielded into a major orange-colored phenoxazine class pigment, which was characterized and identified as 1,2-dimethoxy-3*H*-phenoxazin-3-one (C_14_H_11_NO_4_, M.W. 257), based on UV-Vis, FTIR, HRMS, and NMR analysis. Although this pigment was already described from fungus *A. viticola,* this is the first study reporting the phenoxazine class of pigment from fungus *G. triuniae*. Considering the previous studies describing dyeing potential and bioactivity of phenoxazines, we may finally conclude that the orange pigment “1,2-dimethoxy-3*H*-phenoxazin-3-one” is a promising colourant and possible bioactive compound of *G. triuniae,* having future applications in the textile and pharmaceutical industry.

## Figures and Tables

**Table 1:** The zone of inhibition of test organisms against sample and standards with control.

**Samples**	**The Diameter of the Zone of Inhibition in Millimeters** **(Values are Average of Three Readings)**					
	***E. coli* MTCC 739**	***B. subtilis*** **MTCC 121**	***P. aeruginosa*** **MTCC 2453**	***S. aureus*** **MTCC 2940**	***R. planticola*** **MTCC 530**	***M. luteus*** **MTCC 2470**
Hexane extract of *G. triuniae* NFCCI 4873	-	17.33 ± 1.53	-	18.67 ± 0.58	12.33 ± 0.58	17.33 ± 1.53
Streptomycin	23.33 ± 2.89	18 ± 0	9 ± 0	-	19.67 ± 0.58	38.66 ± 1.15
Ampicillin	18.33 ± 2.89	30.67 ± 2.08	-	14.33 ± 1.15	13.67 ± 0.58	54 ± 0
Chloramphenicol	23.33 ± 2.89	29 ± 1.00	-	26 ± 1.00	19 ± 1.00	26.67 ± 1.53
Ciprofloxacin hydrochloride	43.33 ± 2.89	42 ± 1.00	40 ± 0	34 ± 1.00	30.33 ± 0.58	36.67 ± 1.53
Vancomycin hydrochloride	-	23.33 ± 0.58	-	20 ± 1.00	18.67 ± 0.58	26.67 ± 0.58
Milli-Q water	-	-	-	-	-	-
Methanol	-	-	-	-	-	-

**Table 2:** Compounds identified from hexane extract by GC–MS analysis.

No.	Compound Name	Retention Time	Molecular Weight	Relative Content (%)
1	Undecane	8.361	156	0.34
2	Unknown	17.817	283	0.21
3	1,2-benzenedicarboxylic acid, bis(2-methylpropyl) ester	18.243	278	0.40
4	alpha-D-Mannopyranose, 5TMS derivative	18.594	540	0.19
5	Dibutyl phthalate	18.717	278	0.98
6	n-Hexadecanoic acid	19.116	256	3.56
7	Dibutyl phthalate	19.189	278	0.75
8	Phthalic acid, butyl nonyl ester	19.386	348	0.30
9	alpha-D-Mannopyranose, 1,2,3,4,6-pentakis-*O*-(trimethylsilyl)-	19.459	540	0.28
10	Hexadecanoic acid, trimethylsilyl ester	19.854	328	1.75
11	9-Octadecenoic acid, methyl ester, (*E*)-	20.401	296	0.21
12	9-Octadecenoic acid, (*E*)-	20.801	282	17.62
13	Octadecanoic acid	20.989	284	2.85
14	Unknown	21.380	355	0.25
15	9-Octadecenoic acid, (*E*)-, TMS derivative	21.422	354	1.37
16	9,12-Octadecadienoic acid (*Z*,*Z*)-	21.550	280	0.47
17	Unknown	21.655	327	1.13
18	Hexatriacontane	21.747	506	2.71
19	Unknown	21.820	383	0.51
20	Glycidyl palmitate	22.149	312	0.53
21	Unknown	22.355	340	0.65
22	Unknown	22.697	355	28.08
23	Unknown	22.854	411	30.35
24	Unknown	23.126	397	2.97
25	9-octadecenoic acid, 1,2,3-propanetriyl ester,	23.990	884	1.56

**Table 3:** The ^1^H NMR (500 MHz, CDCl_3_) and ^13^C NMR (125 MHz, CDCl_3_) spectroscopic data for 1,2-dimethoxy-3*H*-phenoxazin-3-one isolated from *G. triuniae* NFCCI 4873 along with the reported one [25].

Position	1,2-dimethoxy-3*H*-phenoxazin-3-one from *G. triuniae* NFCCI 4873		1,2-dimethoxy-3*H*-phenoxazin-3-one Reported from *A. viticola*	
	*δ*_C_	*δ*_H_ (*J* in Hz)	*δ*_C_	*δ*_H_ (*J* in Hz)
1	145.2 C	-	145.1 C	-
2	145.8 C	-	145.9 C	-
3	181.8 C=O	-	181.8 C=O	-
4	104.6 CH	6.23, s	104.7 CH	6.23, s
4a	147.2 C	-	147.3 C	-
5a	143.4 C	-	143.5 C	-
6	115.9 CH	7.33, dd (8.1, 1.07)	116.0 CH	7.33, dd (8.2)
7	132.2 CH	7.54, td (7.7, 1.37)	132.2 CH	7.53, td (8.2)
8	125.3 CH	7.39, td (7.71, 1.37)	125.3 CH	7.39, td (8.2)
9	130.3 CH	7.92, dd (7.93, 1.53)	130.3 CH	7.92, dd (8.2)
9a	132.6 C		132.7 C	-
10a	147.2 C		147.8 C	-
1-OMe	62.2 CH_3_	4.12, s	62.3 CH_3_	4.12, s
2-OMe	61.1 CH_3_	4.14, s	61.2 CH_3_	4.14, s

**Table 4:** GenBank accession numbers of taxa used for phylogenetic analysis.

Sr. No.	Fungal Culture	Strain	GenBank Accession No.	
			ITS	LSU
1	*Gonatophragmium triuniae*	NFCCI 4873	MW193329	MW144438
2	*Gonatophragmium triuniae*	CBS 138901	NR_137932	NG_058117
3	*Gonatophragmium epilobii*	CBS 122271	MH863183	MH874728
4	*Acrospermum* sp.	SGSF153	MK335823	MK754265
5	*Acrospermum longisporium*	MFLU 17-2849	-	NG_064506
6	*Acrospermum gramineum*	M152	-	EU940085
7	*Acrospermum compressum*	M151	-	EU940084
8	*Pseudovirgaria grisea*	CPC 19130	JF957607	JF957612
9	*Pseudovirgaria hyperparasitica*	CPC 10702	EU041765	EU041822
10	*Pseudovirgaria hyperparasitica*	CPC 10704	EU041766	EU041823
11	*Pseudovirgaria hyperparasitica*	CPC 10753	EU041767	EU041824
12	*Pseudovirgaria grisea*	CPC 19126	JF957605	JF957610
13	*Pseudovirgaria grisea*	CPC 19128	JF957606	JF957611
14	*Dyfrolomyces sinensis*	MFLU 17-0777	-	NG_064507
15	*Dyfrolomyces sinensis*	MFLUCC 17-1344	-	MG836699
16	*Dyfrolomyces tiomanensis*	NTOU3636	-	KC692156
17	*Dyfrolomyces rhizophorae*	JK 5456A	-	GU479799
18	*Dyfrolomyces thamplaensis*	MFLUCC 15-0635	-	KX925435
19	*Eremomyces bilateralis*	CBS 781.70	NR_145364	NG_059206

## Data Availability

Not applicable.

## Supplementary Materials

FTIR (Figure S1), HR-MS (Figure S2), ^1^H NMR (Figure S3), COSY (Figure S4), ^13^C NMR (Figure S5), and DEPT-135 (Figure S6) spectra for compound 1,2-dimethoxy-3*H*-phenoxazin-3-one and GC-MS chromatogram (Figure S7) of hexane extract (PDF).
