# Supplementary material for: Antioxidant, Antibacterial and Dyeing Potential of Crude Pigment Extract of Gonatophragmium triuniae and Its Chemical Characterization
Source: Molecules. 2022 Jan 8;27(2):393. doi: 10.3390/molecules27020393 (PMC8779394; doi:10.3390/molecules27020393)

List of Contents

Figure S1. The IR spectrum of 1,2-dimethoxy-3*H*-phenoxazin-3-one.

Figure S2. HRMS spectrum of 1,2-dimethoxy-3*H*-phenoxazin-3-one.

Figure S3. ^{1}H NMR (500 MHz, CDCl3) spectrum of 1,2-dimethoxy-3*H*-phenoxazin-3-one.

Figure S4. COSY (500 MHz, CDCl3) spectrum of 1,2-dimethoxy-3*H*-phenoxazin-3-one.

Figure S5. ^{13}C NMR (500 MHz, CDCl3) spectrum of 1,2-dimethoxy-3*H*-phenoxazin-3-one.

Figure S6. DEPT-135 (CDCl3) spectrum of 1,2-dimethoxy-3*H*-phenoxazin-3-one.

Figure S7. GC-MS chromatogram of crude hexane extract of *G. triuniae* NFCCI 4873.

Figure S1. The IR spectrum of 1,2-dimethoxy-3H-phenoxazin-3-one.

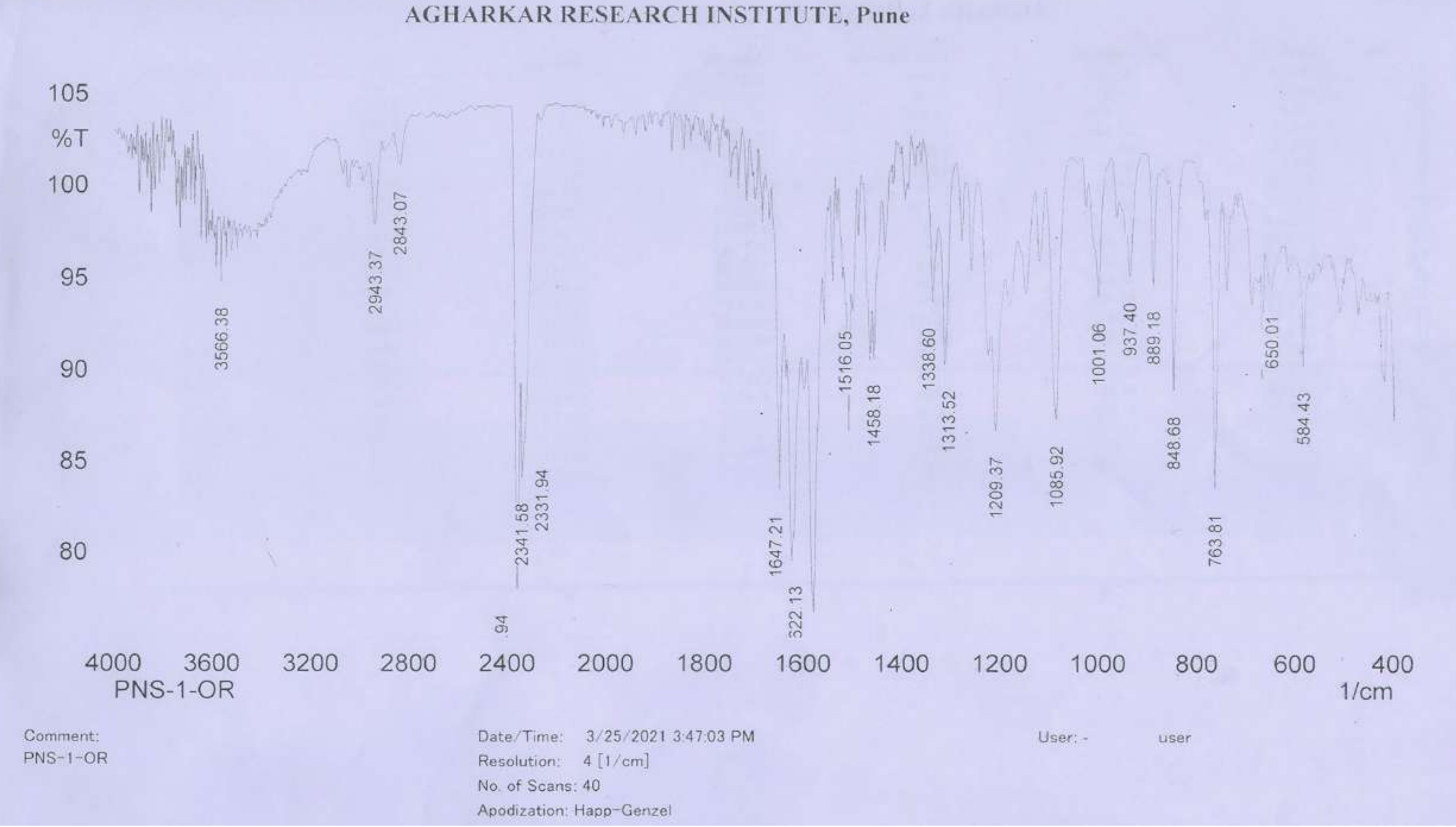

Figure S2. HRMS spectrum of 1,2-dimethoxy-3H-phenoxazin-3-one.

Savitribai Phule Pune University - Central Instrumentation Facility

Analysis Info

Analysis Name	D:\Data\2021\SEP 2021\GOV INST\ARI\DR. S. K. SINGH\AJAY L\PNS-1-OR_GC4_01_3170.d	Acquisition Date	9/2/2021 5:26:53 PM
Method	dlc_ms50-600mz_10min_0.120mlflow_50b.m	Operator	CIF
Sample Name	PNS-1-OR	Instrument	impact HD 1819696.00184
Comment			

Acquisition Parameter

Source Type	ESI	Ion Polarity	Positive	Set Nebulizer	1.7 Bar
Focus	Active	Set Capillary	4500 V	Set Dry Heater	200 °C
Scan Begin	20 m/z	Set End Plate Offset	-500 V	Set Dry Gas	7.0 l/min
Scan End	600 m/z	Set Charging Voltage	2000 V	Set Divert Valve	Source
		Set Corona	0 nA	Set APCI Heater	0 °C

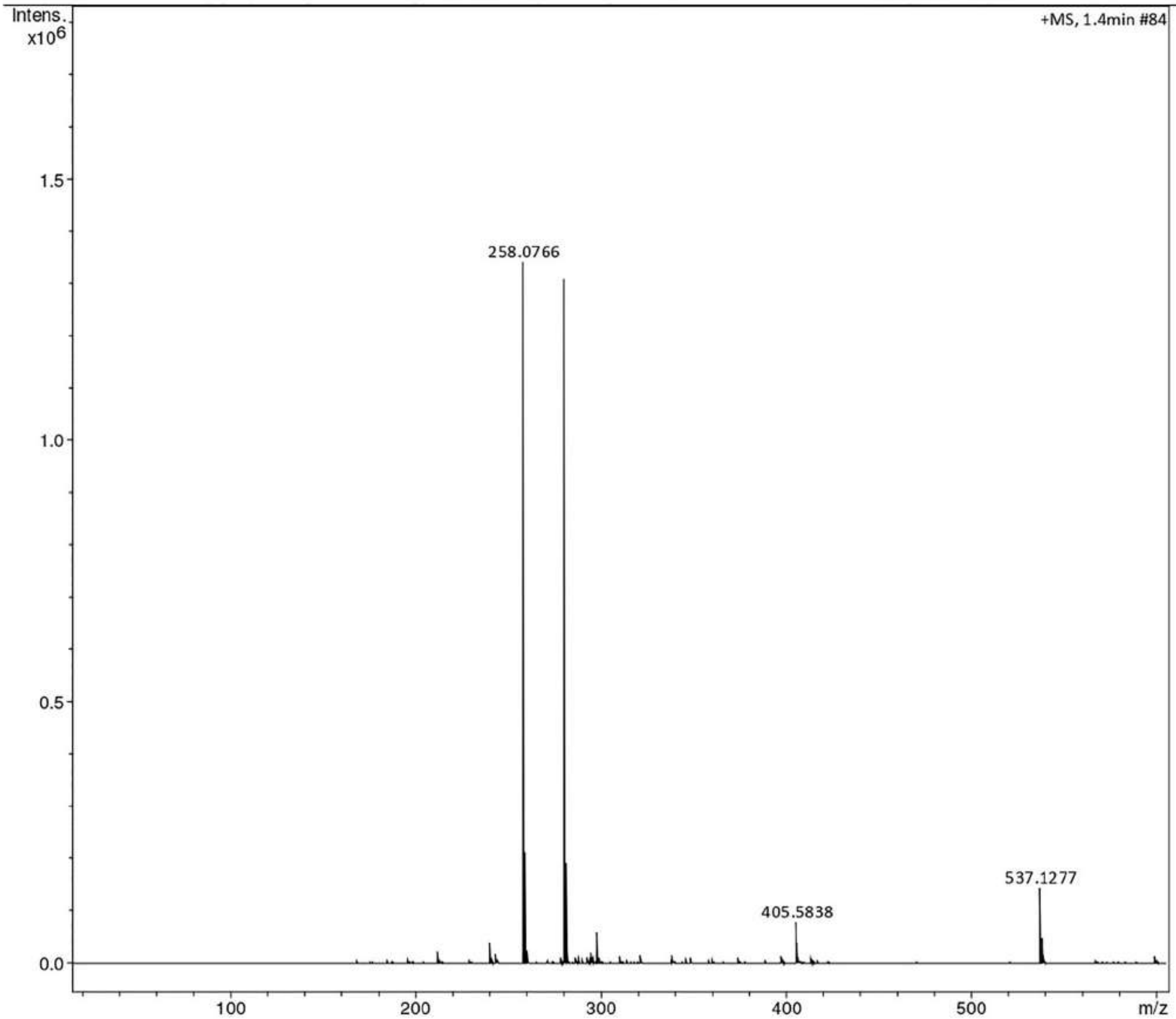

Figure S3. 1H NMR (500 MHz, CDCl3) spectrum of 1,2-dimethoxy-3H-phenoxazin-3-one.

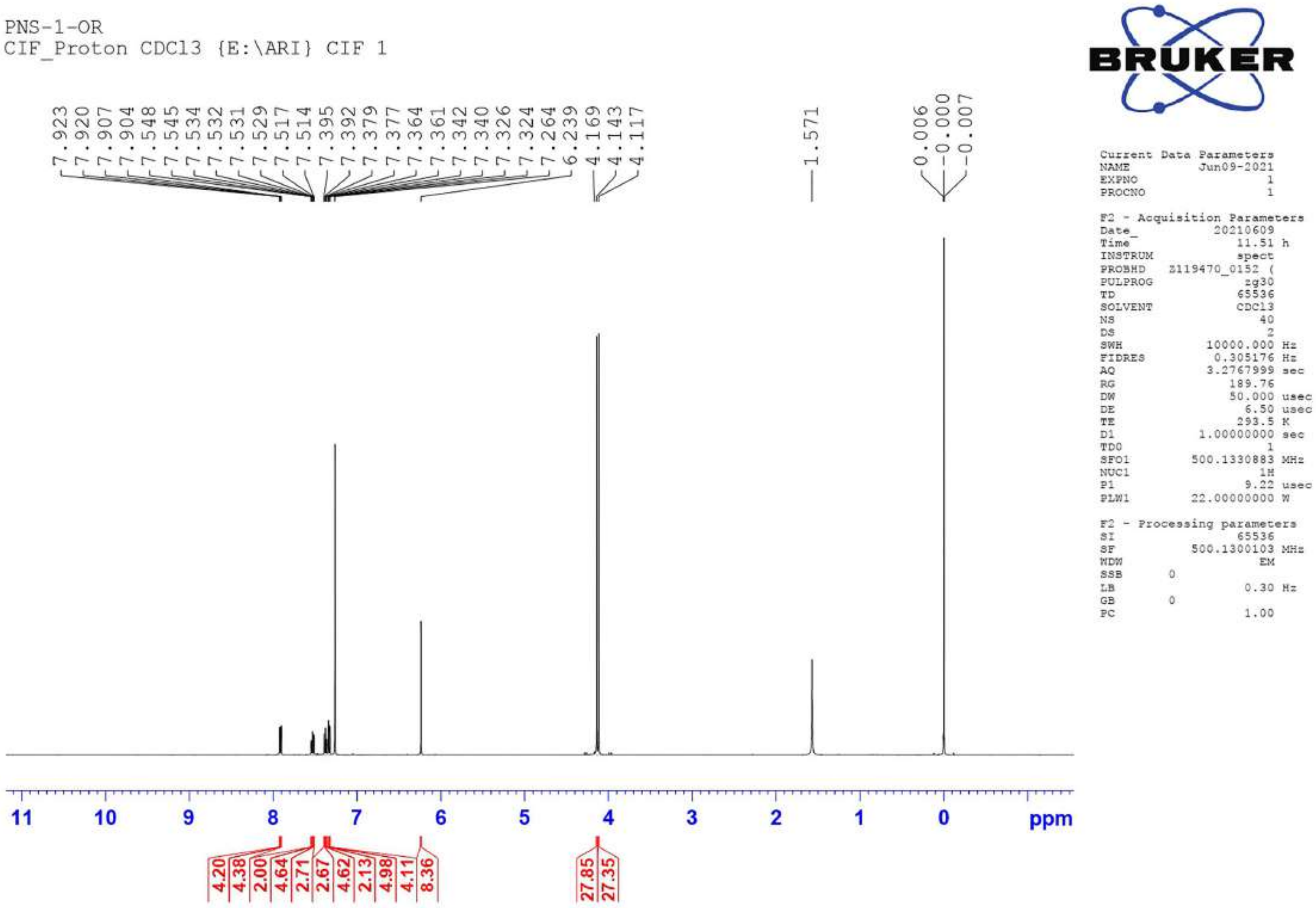

Figure S4. COSY (500 MHz, CDCl3) spectrum of 1,2-dimethoxy-3H-phenoxazin-3-one.

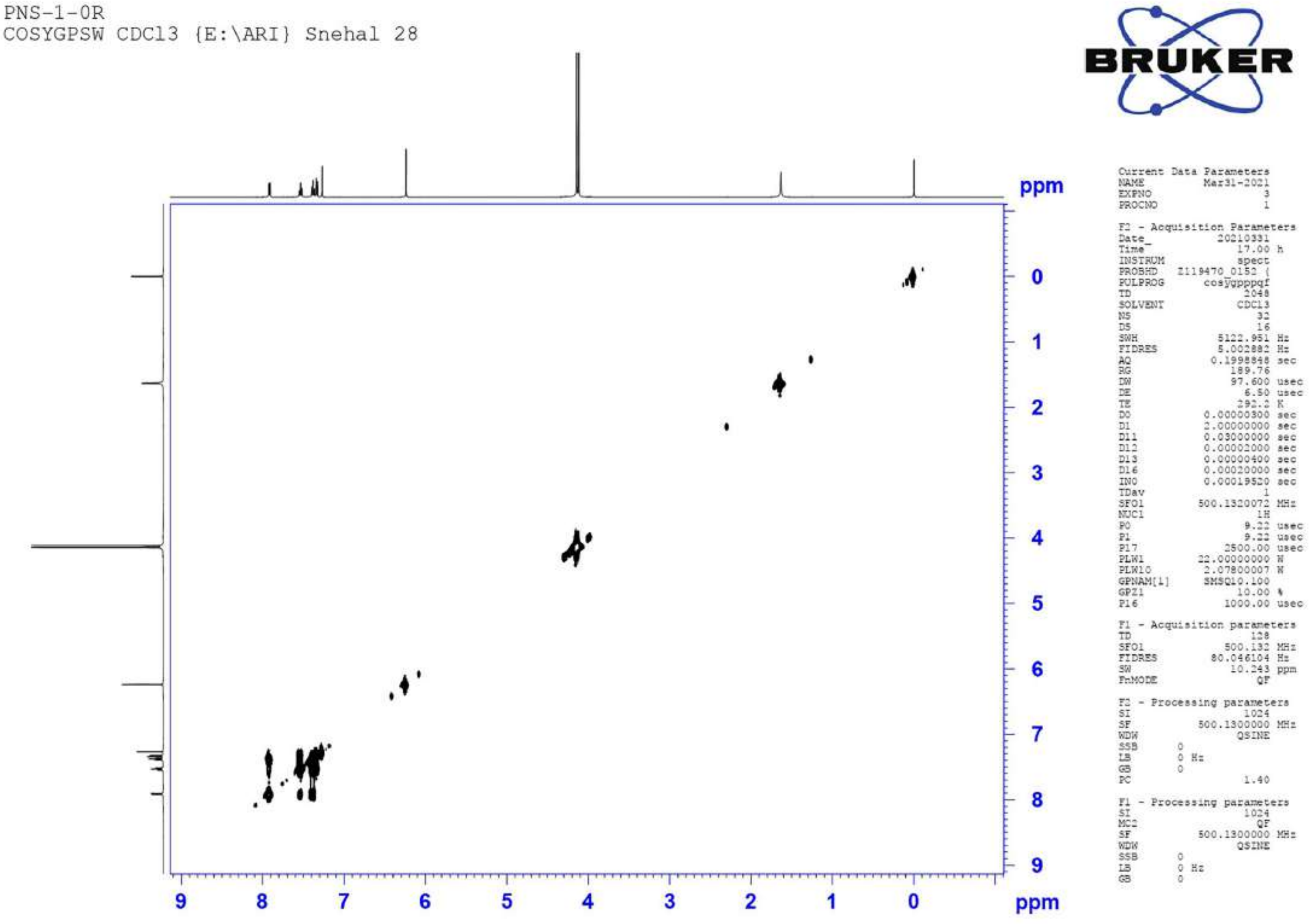

Figure S5. 13C NMR (500 MHz, CDCl3) spectrum of 1,2-dimethoxy-3H-phenoxazin-3-one.

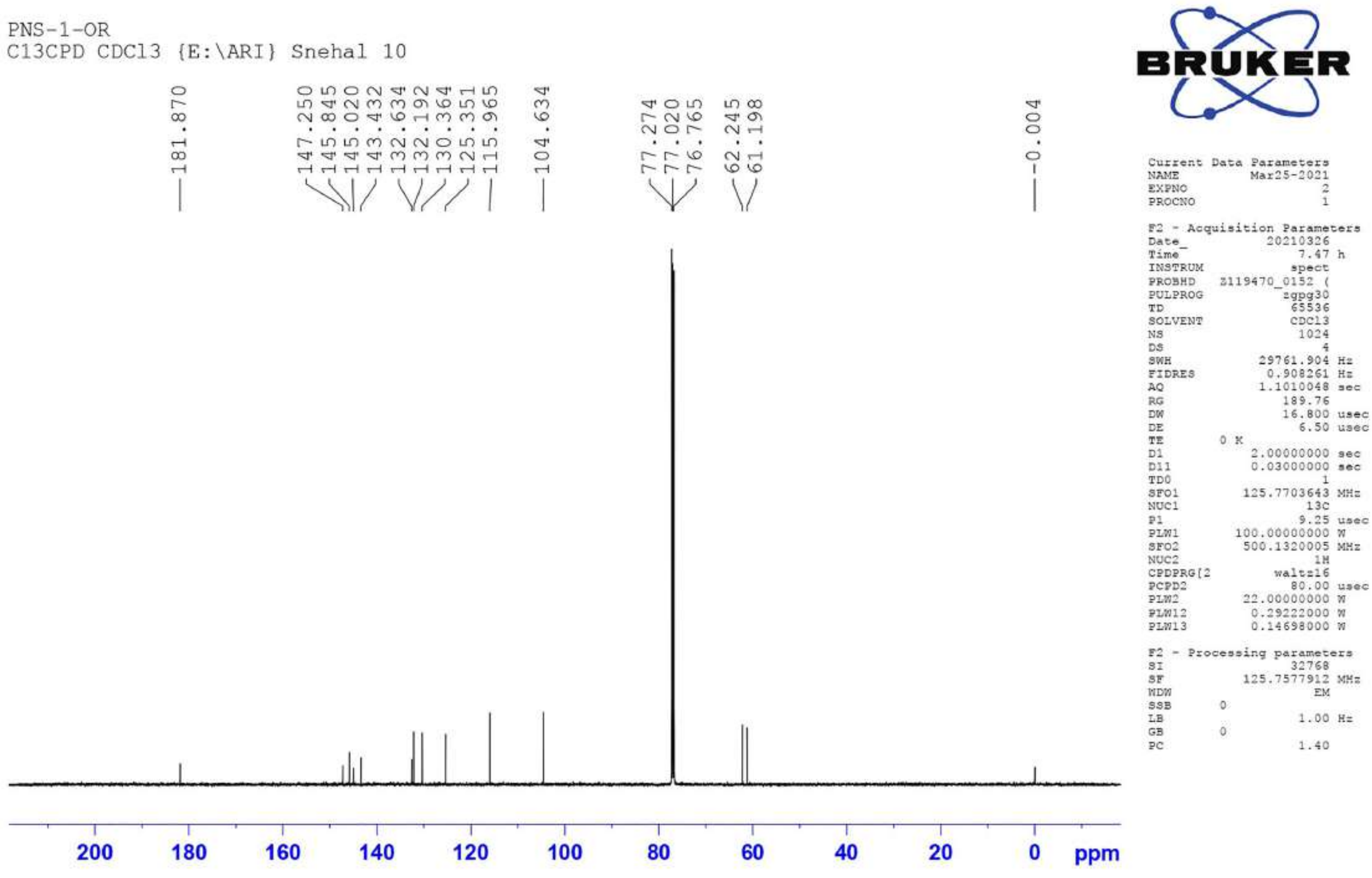

Figure S6. DEPT-135 (CDCl3) spectrum of 1,2-dimethoxy-3H-phenoxazin-3-one.

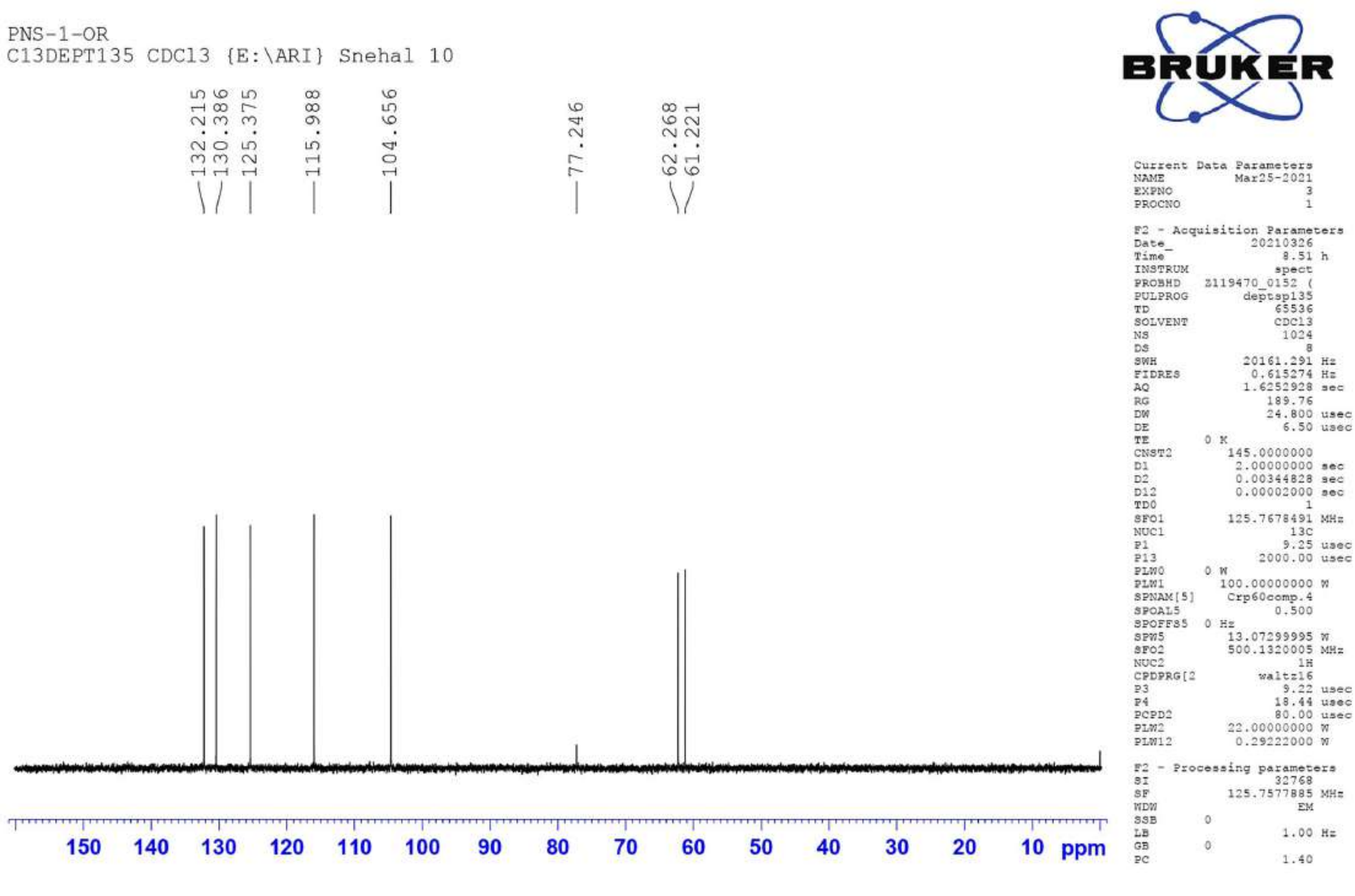

Figure S7. GC-MS Chromatogram of Crude Hexane Extract of *G. truiuniae* NFCCI 4873.

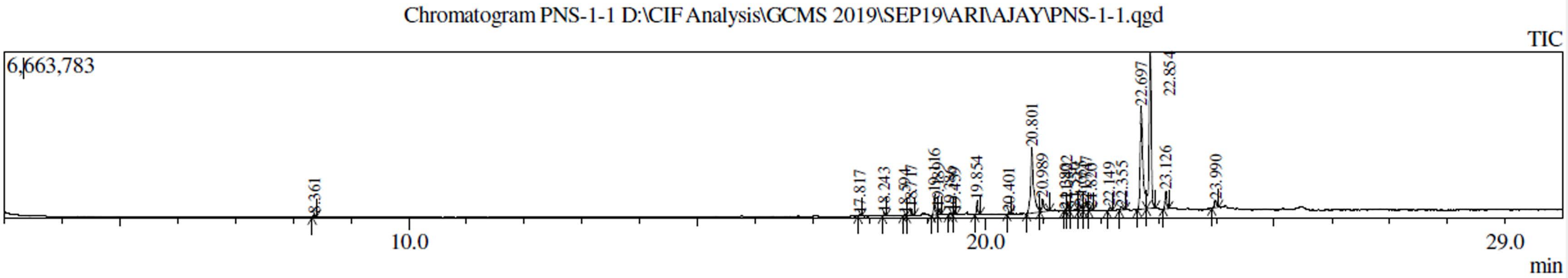

Supplement: Supplementary file 1 [file molecules-27-00393-s001.zip › molecules-1530461-supplementary.pdf]
